# Research on the Influence of Emotional Intelligence and Emotional Labor on the Service Recovery Effect of Online Travel Agency

**DOI:** 10.3389/fpsyg.2021.735756

**Published:** 2021-11-19

**Authors:** Jiahua Wei, Zhiping Hou, Xiaorui Zhou

**Affiliations:** Business School, Guilin University of Technology, Guilin, China

**Keywords:** OTA, service recovery, emotional intelligence, emotional labor, customer loyalty

## Abstract

At present, online travel agency (OTA) service failure events emerge continually, which makes the OTA service operation mode face new challenges. This study uses the situational experiment method to explore the effects of OTA employees’ emotional intelligence and emotional labor (surface behavior and deep behavior) on the effect of service recovery. The results show that the emotional intelligence of OTA employees has a positive impact on the surface behavior and deep behavior; the emotional intelligence and deep behavior of employees have a significant positive impact on service recovery satisfaction, but the positive impact of employees’ surface behavior on service recovery satisfaction is not statistically significant; finally, service recovery satisfaction has a positive impact on customer loyalty. This study helps to better explain the mechanism of OTA service recovery effect and provides a theoretical reference for improving the service recovery effect of OTA.

## Introduction

The operation mode of tourism service under the network environment is undergoing profound changes. With the development of the Internet, great changes have taken place in the display, distribution, and promotion of tourism products, as well as the behavior of consumers (No and Kim, 2015). Due to the huge demand of consumers for online travel, online travel agencies (OTA) have emerged and become a means of consumption (Pinto and Castro, 2019). OTA were first established in 1996 by Expedia (Ling et al., 2014). After years of development, OTA have established a successful economic model and are the online sales channel with the highest reservation rate. Consumers can book tickets, hotels, vacation products and other related tourism products and services from tourism service providers through OTA, and pay online or offline (Xiao, 2021). In essence, OTA are intermediaries that rely on Internet platforms to provide online consulting, commenting, and booking services. Through OTA, consumers can easily compare the products, prices, discounts, independent reviews, and photos of different hotels. Therefore, OTA cater to the needs and preferences of consumers’ diversification, and they have developed rapidly as a result, with the emergence of famous brands such as Priceline, Expedia, and Ctrip.

However, due to the intangible, synchronous, and heterogeneous characteristics of service products, OTA service failure is inevitable in the process of serving consumers. At present, OTA service failure events emerge continually. For example, during the “5.1” holiday in Chongqing, China in 2019, OTA became the “disaster area” of consumer complaints. There are consumer complaints in all aspects of ticket booking, travel, hotel accommodations, and tourist attraction consumption. The service failure complaints include high refund fees, inability to process orders, difficulty in making an appointment after placing an order, rejection of refunds and renewals, no refund for no board, and special-price goods. Therefore, service failure has become the curse of OTA and service providers, such as hotels, and presents new challenges to the OTA service operation mode. Service recovery is the only way for OTA to restore customer satisfaction and enhance competitiveness.

The existing literature has mainly focused on service failure and service recovery. For example, some scholars have constructed a measurement system of online shopping service recovery quality (Liu and Li, 2017) and conducted an empirical study on the relationship between customer participation and customer loyalty in service recovery (Ashraf and Manzoor, 2017); they have also examined the relationships between service failure, service recovery, and customer satisfaction and loyalty (Jian and Ke, 2017). For the service recovery of the tourism industry, academic circles have also studied the impact of word-of-mouth on the service recovery effect of travel agencies. The service recovery effect is measured by customer satisfaction and customer loyalty (Pai et al., 2019). Some scholars have studied the impact of service quality on customer loyalty after service failure in travel agencies (Kuo et al., 2013), developed a practical manual on service failure and service recovery in the travel industry (Inkson, 2019), and studied the impact of the emotional intelligence of travel agency employees on customer orientation and service recovery efforts (In et al., 2016). However, there is still a lack of research on OTA service failure and service recovery in academic circles. It is urgent to explore the mechanism of OTA service recovery.

In the past 20 years, the research on emotional intelligence and emotional labor has attracted the attention of management academia. Combined with vivid management practice, a large number of cross research results have emerged. Some studies have shown that managers’ emotional intelligence has a significant positive impact on job performance through the mediating effect of leadership and management self-efficacy on subjective performance (Zhang et al., 2009). In the field of service management research, it has been found that the emotional intelligence of employees has a positive impact on customer orientation and customer loyalty (Zhan, 2012; In et al., 2016). Some studies have also confirmed that employees’ emotional labor has a significant negative impact on life satisfaction, job satisfaction, and customer satisfaction (Abdul et al., 2008; Gong et al., 2012). However, under the service recovery situation of OTA, the influence mechanism of the emotional intelligence and emotional labor of employees on the service recovery effect is still unclear, and clarifying these problems will help OTA improve the service recovery effect and their corporate image.

This study uses the situational experiment method for empirical analysis and introduces the concept variables of emotional intelligence and emotional labor into the service recovery effect of OTA. It aims to examine the relationship between emotional intelligence and emotional labor, as well as their relationships with service recovery satisfaction, and explore the mechanism of customer loyalty after service recovery. This study will try to expand the horizons of service recovery research and OTA research, as well as expand the application of emotional intelligence and emotional labor in the field of management, highlighting the advantages of cross research, so as to better explain the impact mechanism of OTA service recovery effect. In practice, this study will focus on improving the service recovery effect and capacity of OTA, promoting the healthy development of OTA business model, and better serving tourists all over the world.

## Literature Review and Research Hypothesis

### Online Travel Agency

The development of new technologies and the Internet has changed the way tourists book their accommodations, leading to the development of online booking channels, including OTA (Rianthong et al., 2016). OTA were first established by Expedia (Ling et al., 2014) in 1996 and subsequently developed rapidly throughout the world. OTA provide an effective platform for consumers to browse and purchase travel services and share travel information through websites, mobile devices, apps, and call centers. OTA are a tool for marketing, searching, and booking and can earn tourism market share by managing hotel room reservations (Sheng, 2019). In other words, OTA are an intermediary that provides online consultation, comment, and reservation services. The emergence of OTA is a phenomenon that affects traditional travel agencies and poses an important challenge to them (Fahlevi, 2021). Because OTA have changed the way of tourism purchase, catered to the needs of consumers, and have great development potential, traditional tourism agencies continue to strengthen cooperation with them to meet the needs of consumers (Sheng, 2019).

For hotels, OTA help increase visibility, thus increasing the interest and occupancy rate of tourists (Ling et al., 2015). OTA have played an important role in building the reputation of hotels. One of the factors contributing to the success of Weiganghui Hotel in Hong Kong was the close cooperation with OTA at the beginning of its operations and the expansion of its promotion scope (Tony, 2019). However, Green and Lomanno (2012) believes that OTA generate little value, sharing the revenue that should belong to the hotel and not creating value.

### Service Failure and Service Recovery

Service failure refers to that the products or services produced by enterprises fail to meet the needs of customers or fail to reach the level expected by customers (Huang, 2021). A large number of studies have shown that service failure leads to serious negative consequences, such as customer dissatisfaction, negative word-of-mouth publicity, and turning to competitors (Liu et al., 2021). The academic community generally divides service failure into result failure and process failure. Result failure mainly refers to the service provider failing to achieve the basic service content and meet customer expectations, while process failure mainly refers to an unpleasant service experience in the service delivery mode (Bitner et al., 1990).

After the service failure occurs, in order to avoid or reduce the loss of image, the enterprise needs to carry out service recovery. Service recovery is an action taken against service failure, and it involves the enterprise’s hard work to make the service meet the customer’s expectations (Zhong et al., 2011). At present, the discussion of service recovery can be divided into macro and micro levels. Service recovery at the macro level is the overall management process of the service quality system, while service recovery at the micro level is the immediate and active response measures taken by enterprises to customers. The common recovery measures include apology, explanation and compensation (Li and Li, 2021). Scholars have paid extensive attention to the influencing factors of the service recovery effect and examined the relationship of the perceived fairness of online shopping, the retail industry, and the tourism industry with service recovery quality, customer satisfaction, and customer loyalty (Assefa, 2014). Studies have also investigated the relationship among customer participation, joint remedy, and the service recovery effect and suggested that customer participation and joint remedy improve the service recovery effect (Ashraf and Manzoor, 2017).

On the whole, the current theory of service failure and service recovery is relatively mature, but there is insufficient attention to OTA service failure and service recovery, and the relevant literature is still lacking, which is difficult to explain and guide the current OTA service failure and service recovery practice. Therefore, the current research on service failure and service recovery should pay attention to emerging service fields, such as OTA, and constantly expand the breadth and applicability of theory to better guide practice.

### Emotional Intelligence and Emotional Labor

Emotional intelligence is a kind of intelligence related to emotion, which emphasizes the guiding ability of perception, integration, understanding and management of emotional information in individual behavior (Luo et al., 2021). At present, there are two typical ways to measure emotional intelligence: Task-based and questionnaire based. Task-based is based on the right and wrong of solving problems, while questionnaire based is based on the evaluation level (Pu et al., 2017). As for the impact of emotional intelligence, scholars have paid continuous attention to the impact of employees’ emotional intelligence on organizational performance, work attitude and behavior (Mubeen et al., 2016; Sajjad et al., 2017). Based on the Chinese cultural context, Wong and Law (2002) developed an emotional intelligence scale in the Chinese context. The scale consists of four parts: Self emotional assessment, others’ emotional assessment, emotional regulation, and emotional utilization.

Emotional labor is a process in which employees, for the benefit of the organization and at the cost of consuming positive emotions, strive to play the professional role expected by customers in exchange for remuneration (Ni and Cui, 2021). Emotional labor can be divided into two aspects: surface behavior and deep behavior. Surface behavior refers to the work that employees do to adjust the visible aspects of emotion, such as voice and facial expression, to meet the emotional expression requirements of the organization on the surface when the emotion and performance rules of employees are inconsistent. Deep behavior refers to work that changes internal thoughts and emotions to make employees’ inner cognition and organizational needs consistent (Brotheridge and Grandey, 2002). Chi et al. (2018) believed that emotional labor of front-line service personnel is an important way to improve customer experience and emotional value. Academic circles have also studied the impact of employees’ emotional intelligence on emotional labor, and believe that there is a positive correlation between emotional intelligence and emotional labor, and there is a positive impact relationship (Lu et al., 2016). Chen et al. (2010) believed that there was a positive correlation between police emotional intelligence and emotional labor, and the positive effect of emotional intelligence on deep behavior was greater than that on surface behavior and natural behavior. Prior research from several different service industries has found that service employees’ emotional intelligence positively affects surface behavior and deep behavior, and deep performance plays a partial intermediary role between emotional intelligence and organizational citizenship behavior (Yang et al., 2013). Prior research have further confirmed that under the traditional service recovery scenario, service employees’ emotional intelligence has a positive impact on emotional labor (Wei, 2016). Compared with the traditional service recovery scenario, employees’ emotional intelligence and emotional labor ability under OTA service recovery scenario have not changed greatly, and employees’ emotional intelligence will also affect surface behavior and deep behavior. Based on the above analysis, this paper puts forward the following two research hypotheses:

H1: Emotional intelligence has a positive effect on surface behavior.

H2: Emotional intelligence has a positive effect on deep behavior.

### Service Recovery Satisfaction

Customer satisfaction was first applied to the field of management by Cardozo in 1965, and has been a hot issue in service research in the past 50 years. At present, the definition of customer satisfaction mainly includes two perspectives. The specific perspective on customer satisfaction holds that customer satisfaction is an immediate emotional reflection of the customer’s satisfaction with the value obtained after consumption in a specific situation, while the overall perspective on customer satisfaction considers customer satisfaction as a holistic attitude based on experience and formed by the customer’s attitude (Woodside et al., 1989). This study adopts a specific perspective to define customer satisfaction, considering service recovery satisfaction as an emotional state in which the actual perceived effect of instant service recovery is higher than the expected service recovery. Its most prominent feature is customer satisfaction in a specific situation of service recovery, which is a kind of “second-degree satisfaction.”

In the medical field, it is found that nurses’ emotional intelligence is positively correlated with patient satisfaction (Zhang et al., 2014). Rozell et al. (2006) found that the emotional intelligence of salesmen has a significant impact on their sales performance. Furthermore, the surface behavior of employees can repel customers. A fake smile cannot move customers, while a sincere display of positive emotion can significantly improve customer satisfaction (Zhang, 2011). In the tourism industry, some scholars have found that employee emotion assessment and emotion regulation have a positive impact on customer satisfaction (Zhan, 2012). Wei (2016) based on the traditional service recovery scenario, has a positive impact on employees’ emotional intelligence and emotional labor on service recovery satisfaction. However, there is still a lack of relevant research on the relationship between emotional intelligence and emotional labor on service recovery satisfaction in OTA service recovery scenario. In the OTA service recovery scenario, the psychological status of employees participating in service recovery will also be similar to that of employees in the traditional service recovery scenario. Employees with high emotional intelligence are more likely to improve service recovery satisfaction, because employees with high emotional intelligence can better control and manage their emotions. The superficial behavior of employees, such as hypocrisy and external smile, will cause customer disgust and reduce service recovery satisfaction, while the deep behavior of employees will improve service recovery satisfaction. Therefore, the following research hypotheses can be proposed:

H3: Emotional intelligence has a positive impact on service recovery satisfaction.

H4: Surface behavior has a negative impact on service recovery satisfaction.

H5: Deep behavior has a positive impact on service recovery satisfaction.

### Customer Loyalty

Early research often defined customer loyalty as a customer’s positive psychological attachment to a certain product or service and repeat purchase behavior (Fornel, 1992). Later, the academic community thought that it was not comprehensive to define customer loyalty by psychological attachment and repeat purchase. At present, the academic community has generally accepted to define customer loyalty from two perspectives: Behavior loyalty and attitude loyalty (Yu et al., 2008). Chen et al. (2015) believes that customer loyalty refers to customers’ choice preference and repeated purchase behavior for enterprise products or services over a long period of time. In terms of customer loyalty dimension research, customer loyalty can be divided into a cognitive component, an emotional component, reconstruction intention, repeat purchase, recommendation to others, and attention (Li, 2014). Through an empirical analysis of the Beijing service industry, Lu (2007) found that customer satisfaction has a direct positive impact on customer loyalty. Although the above research results do not involve the situation of OTA service recovery, they have important reference value for this study. In the scenario of OTA service recovery, when customers are satisfied with service recovery, they will recommend and repeat purchases to the surrounding people, resulting in behavior loyalty and attitude loyalty. Therefore, the following research hypotheses are proposed:

H6: Service recovery satisfaction has a positive impact on customer loyalty.

From the above analysis and hypothetical relationship, we can see that under the OTA service recovery scenario, the relationship between the emotional intelligence of employee and customer loyalty plays a role through the service recovery satisfaction, and the service recovery satisfaction has a mediating effect on the relationship between emotional intelligence and customer loyalty. The emotional intelligence of employees also has an impact on customer loyalty through surface behavior and service recovery satisfaction. Surface behavior and service recovery satisfaction play a mediating role in the relationship between emotional intelligence and customer loyalty. At the same time, under the OTA service recovery scenario, the emotional intelligence of employees also has an impact on customer loyalty through two variables: deep behavior and service recovery satisfaction. Therefore, the following three hypotheses are put forward.

H7: Service recovery satisfaction plays a mediating role in the relationship between emotional intelligence and customer loyalty.

H8: Surface behavior and service recovery satisfaction plays a mediating role in the relationship between emotional intelligence and customer loyalty.

H9: Deep behavior and service recovery satisfaction plays a mediating role in the relationship between emotional intelligence and customer loyalty.

**FIGURE 1:**
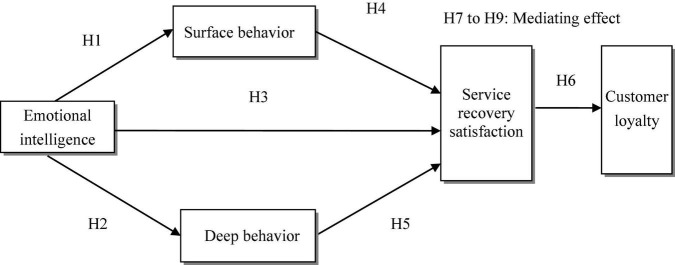
Research model.

Based on the above nine hypotheses, a research model is proposed, as shown in Figure 1.

## Research Design

### Experimental Design

In recent years, although service failures of OTA often occur, due to the limitation of time and space, it is very difficult for tourism researchers to investigate and study service failures and service recovery. In order to improve the pertinence and effectiveness of the research and provide research support for the subsequent research hypothesis demonstration, this study refers to the practice of Fang et al. (2019), Cheng and Li (2020), and adopts the method of situational experiment to collect and study data on the basis of real cases. In order to improve the external validity and pertinence of the experiment, the experimental materials are from OTA service failure events reported by Chinese media, and refer to the tourism complaints published by China National Tourism Administration.

The experiment simulates that consumers encounter OTA service failure, and the OTA take service recovery. In the experiment, all subjects were randomly divided into three groups, each set in a scenario. In each group, 50% of the subjects acted as OTA service recovery employees, and 50% of the subjects acted as OTA customers. In the experiment, the subjects were asked to read the text description of their situation carefully and put the individual in the scene. The researcher explained various scenarios and guided the subjects to imagine their feelings and reactions as OTA employees or consumers under the situation of OTA service failure and service recovery. Then, the researcher instructed the subjects to fill in the questionnaire of the situational experiment.

Scenario 1: After service failure, OTA immediately cooperate with hotels or scenic spots to take the initiative to carry out service recovery. The employees participating in the service recovery have high emotional intelligence, have good insight into consumers’ emotions and psychology, and behave appropriately in surface and deep-seated behaviors. On behalf of the OTA, the employees apologize sincerely and take recovery measures such as refund of fees, monetary compensation, service product discount, and replacement of service items. Consumers are more satisfied with service recovery, which increases their trust in OTA, and they will continue to purchase the services from OTA in the future.

Scenario 2: After service failure, OTA will immediately cooperate with hotels or scenic spots to take the initiative to carry out service recovery. The employees’ performance and service recovery measures are the same as in scenario 1. However, consumers have high expectations for OTA service recovery and are still not satisfied with the existing service recovery work. They will not purchase the OTA service products again.

Scenario 3: After service failure, OTA immediately cooperate with hotels or scenic spots to take the initiative to carry out service recovery. However, the emotional intelligence of employees participating in service recovery is relatively low, and they cannot have good insight into consumers’ emotions and psychology. The surface behavior and deep behaviors of employees are not appropriate, they do not offer a sincere apology, and they may even hurt the consumers again. OTA fail to implement timely service recovery measures such as refund of fees, monetary compensation, and replacement of service items. Consumers are not satisfied with the service recovery work of the OTA, and their trust in OTA is reduced. They will not continue to purchase OTA tourism services in the future.

### Variable Measurement

There are five measurement variables in this study, of which surface behavior and deep behavior are two variables to measure employees’ emotional labor. In this study, the relevant scales of authoritative literature were used and modified according to the service recovery scenarios of OTA. We used the eight-item WLEIS of Wong and Law (2002) to measure emotional intelligence. Surface behavior and deep behavior are both variables to measure emotional labor. Therefore, referring to Chen’s (2010) emotional labor scale, four items were selected to measure surface behavior and deep behavior. The measurement of service recovery satisfaction was based on the customer satisfaction scales of Bhatta and Premkumar (2004), Ribbink et al. (2004) and combined with the characteristics of service failure and service recovery for five total items. The measurement of customer loyalty was based on the customer loyalty scale compiled by Zeithaml et al. (1996), and four items were selected, including the two aspects of customer behavior loyalty and customer attitude loyalty. We invited 12 citizens with OTA service experience and 5 service management researchers to revise the text content and expression of the first draft of the questionnaire, and corrected the ambiguous meaning and unclear expression in the questionnaire.

The questionnaire was divided into two parts: an employee part and a customer part. The employee questionnaire included the three variables of emotional intelligence, surface behavior, and deep behavior, with a total of 16 items. The customer part of the questionnaire included the two variables of service recovery satisfaction and customer loyalty, with a total of nine items. All of the items were measured using a five-point Likert scale with the options “very consistent,” “consistent,” “basically consistent,” “not very consistent,” and “not consistent.”

### Pre-experiment

The purpose of the pre-experiment was to test the authenticity of the situation and the manipulation of variables. In the pre-experiment, a group of college students with high internal consistency was used because college students are an active group of OTA consumers that have a good reference value. A total of 54 undergraduates and postgraduates were randomly assigned to scenario 1, scenario 2, and scenario 3, with 18 participants in each group. In each group, nine people acted as OTA’ service recovery employees and nine as OTA customers. In the pre-experiment, the subjects were asked to read the text description of their own situation carefully and place themselves in the scene. The researcher explained various scenarios and guided the subjects to imagine their feelings and reactions as OTA employees or consumers in the context of OTA service failure and service recovery. Then, the subjects were instructed to fill in the questionnaire. The participants who played the role of employees filled in the employee questionnaire, and the subjects who played the role of customers filled in the customer questionnaire.

After the questionnaire survey, the researchers conducted an independent sample *t* test on the questionnaire data. The results showed that there was a significant difference in emotional intelligence between the high emotional intelligence group and the low emotional intelligence group (control group) (*M*_*non–confirming*_ = 2.28, *M*_*confirming*_ = 3.66, *t* = 3.704, df = 49, *P* < 0.001), and emotional intelligence manipulation passed the test. Compared with the control group, there was a significant difference in the surface behavior between the group with good surface behavior and the group with poor surface behavior (*M*_*non–confirming*_ = 2.21, *M*_*confirming*_ = 3.86, *t* = 2.371, df = 38, *P* < 0.005), and the manipulation of surface behavior passed the test. We also tested the manipulation of deep behavior, and the findings showed that there was a significant difference in deep behavior between the high deep behavior group and the low deep behavior group (control group) (*M*_*non–confirming*_ = 2.33, *M*_*confirming*_ = 3.92, *t* = 5.610, df = 38, *P* < 0.001). The results of the independent sample *t* test of the above pre- experiments showed that emotional intelligence and emotional labor (surface behavior and deep behavior) were successfully manipulated.

### Formal Experiment

The formal experiment involved the researchers, 2 students, and 15 community and village committee staff. In December 2019, a formal experiment was conducted on a sample of citizens from Guilin and Hezhou in China and farmers from Yangshuo County of Guilin. The formal experiment collected sample data through the following channels. One was to cooperate with the community of Guilin (the grassroots management organization of Chinese cities) and collect experimental samples through the community’s social platforms (QQ group and WeChat group) and require the subjects to have OTA service purchase experience. Second, with the improvement of China’s rural economic development level, nine-year compulsory education has been popularized in rural China, and the cultural level has been improved rapidly. Therefore, we cooperated with the village committee of Yangshuo County, Guilin (the grassroots management organization in rural China), to collect experimental samples through the social platform (QQ group) of the village committee and required the subjects to have OTA service purchase experience.

Due to the different living areas of the samples, the experiment was completed six times. In each experiment, we divided the experimental samples into an employee group and a consumer group of equal size. Then, the participants were asked to choose the most similar scenario and fill in the questionnaire according to their own OTA service purchase experience. In order to improve the quality of questionnaire filling, the researchers gave necessary guidance to the subjects. At the same time, in order to ensure the filling effect and respect personal privacy, subjects were allowed to complete the filling in a free private space as far as possible.

In order to improve the quality of questionnaire data, researchers try to avoid non- response bias during the experiment. Academia believes that in the questionnaire survey, there are many factors that will lead to the lack or non-response of some individuals in the sample, thus affecting the data quality of the questionnaire (Smironva et al., 2019). Therefore, the following measures were taken to avoid the problem of non-response bias. First of all, in the experiment, the subjects voluntarily participated in the situational experiment, and the samples were required to have OTA service purchase experience, which greatly reduced the subjects’ unresponsive response. Secondly, before the experiment, the researchers conducted the necessary training for the subjects, focusing on the detailed introduction of the experimental operation process and the questionnaire filling and answering methods. Thirdly, at the scene of the situational experiment, the researchers checked the questionnaires submitted by the subjects one by one, and re filled in the questionnaires that did not meet the requirements.

In the formal experimental stage, a total of 346 questionnaires were collected. After excluding incomplete questionnaires and those with inconsistent answers, 324 questionnaires were valid, and the effective rate was 93.64%. Because the questionnaire was divided into simulated employees (emotional intelligence, surface behavior, and deep behavior) and simulated consumers (service recovery satisfaction, customer loyalty), the sample number of service employees and customers was the same, 324. The respondents included civil servants, teachers, enterprise staff, doctors, students, farmers, and other professions. In the sample of simulated employees, 166 were male and 158 were female, accounting for 51.23% and 48.77%, respectively; 239 had a college degree or above, accounting for 73.77%; 189 were 18–35 years old, accounting for 58.33%. In the sample of simulated consumers, 152 were male and 172 were female, accounting for 46.91% and 53.09%, respectively; 221 had a college degree or above, accounting for 68.21%; 197 were 18–35 years old, accounting for 60.80%.

## Data Analysis

### Common Method Bias Test

In this study, although the measurement types of five variables such as emotional intelligence, surface behavior and deep behavior are derived from the authoritative scale and measured by Likert 5-point scale, they belong to self-reported questionnaire, there may be common method bias, and common method bias test is needed. Referring to the suggestions of Podsakoff et al. (2003), Tang and Wen (2020), this study decided to use Harman single factor test method to test the common method deviation. This study used SPSS22.0 software for analysis. The results show that there are 25 common factors in this study, and the variance interpretation percentage of the first common factor is 25.77%. Tang and Wen (2020) believe that when the variance interpretation percentage of the first common factor is less than 40%, it indicates that there is no serious common method bias. Therefore, there is no serious common method bias in this study.

### Reliability and Validity Test

Generally, Cronbach’s α coefficient is used for reliability testing. The higher the coefficient value, the higher the reliability. Cronbach’s α greater than 0.70 indicates good reliability (Hair and Black, 2006). In Table 1, the Cronbach’s α values of emotional intelligence, surface behavior, deep behavior, service recovery satisfaction, and customer loyalty were between 0.828 and 0.899, greater than the threshold value of 0.70, indicating that the questionnaire has good reliability.

**TABLE 1:** Test for reliability and convergent validity.

Variables	Items	Normalized load factor	*t-*value	Cronbach’s α	CR	AVE
Emotional intelligence	1. I know my emotions very well	0.799	7.681			
	2. I can quickly detect the customer’s emotion	0.743	4.021	0.867	0.930	0.627
	3. I can always motivate myself	0.726	4.952			
	4. I have strong emotional self-control ability	0.903	9.723			
	5. I know whether I am happy or not	0.731	3.765			
	6. I have the perseverance to achieve the goal	0.823	4.235			
	7. I think I am a capable person	0.784	6.386			
	8. I can deal with crisis rationally	0.807	3.862			
Surface behavior	9. I can show the right expression	0.812	2.996	0.847	0.881	0.650
	10. I will pretend to be in a good mood	0.796	2.231			
	11. I can show the emotions that work needs	0.793	4.036			
	12. I will hide my true feelings	0.824	3.765			
Deep behavior	13. I think about consumers in my heart	0.849	4.034	0.828	0.889	0.667
	14. I can understand consumers in my heart	0.788	5.347			
	15. I will serve customers sincerely	0.795	2.998			
	16. My emotions are from the heart	0.834	6.708			
Service recovery satisfaction	17. I am satisfied with the opportunity of service recovery	0.809	2.872	0.833	0.873	0.576
	18. I am satisfied with the service recovery	0.783	3.871			
	19. Service recovery solves my problem	0.746	4.863			
	20. Service recovery meets my psychological expectation	0.727	6.189			
	21. I am satisfied with the staff’s performance	0.824	4.762			
Customer loyalty	22. I will continue to purchase the OTA’s service	0.807	6.483	0.899	0.916	0.733
	23. I will recommend the OTA	0.872	4.782			
	24. I will be a fan of the OTA	0.817	6.622			
	25. I have increased my sense of belonging to the OTA	0.921	3.879			

*CR represents combined reliability.*

The validity test included a content validity test, convergence validity test, discrimination validity test, and construct validity test. In terms of content validity, all items of the questionnaire were based on the research results of authoritative published journals and were adjusted for OTA service recovery scenarios, which shows that the questionnaire items have good content validity. The convergence validity is shown in Table 1. The standardized load coefficient of each item was greater than 0.50, and the *t*-Value was greater than the threshold value of 1.96. The combined reliability of each variable was greater than 0.70, and the average refined variance (AVE) was greater than 0.50, which conformed to the convergence validity test standard of Hair and Black (2006), Wu (2010), so it passed the convergence validity test.

The discriminant validity analysis is shown in Table 2. The square root values of the AVE of the five variables were greater than the correlation coefficient between the variable and other variables. According to Wu’s (2010) standard, the questionnaire has good discriminant validity, and the discriminant validity passed the test. In terms of construct validity, Amos 22.0 was used for confirmatory factor analysis. The results of confirmatory factor analysis of the overall fit of the model were as follows: χ^2^/DF = 1.782, RMSEA = 0.035, GFI = 0.983, NNFI = 0.961, CFI = 0.971, PNFI = 0.724, and PGFI = 0.761. According to Wu (2010), χ^2^/DF should be less than 5; GFI, CFI, and NNFI should be greater than 0.90; SRMR should be less than 0.05; and RMSEA should be less than 0.1. Therefore, all of the above indicators of the research model met the standard of a good model, and the questionnaire passed the construct validity test.

**TABLE 2:** Test for discriminant validity.

Variables	1	2	3	4	5
1. Emotional intelligence	0.792				
2. Surface behavior	0.637	0.806			
3. Deep behavior	0.665	0.582	0.817		
4. Service recovery satisfaction	0.318	0.294	0.615	0.759	
5. Customer loyalty	0.299	0.214	0.581	0.697	0.856

*The value on the diagonal is the square root of AVE; the other data are the correlation coefficients of the corresponding variables.*

### Direct Impact Relationship Test

Combined with the special of this study, it is decided to use structural equation modeling method to test the research hypothesis relationship between variables. The reasons for adopting the structural equation modeling method in this study are as follows: Because the structural equation model can include multiple dependent variables at the same time, while the regression analysis will ignore the existence and influence of other dependent variables when analyzing the influence relationship on a dependent variable. The structural equation model analysis can give different evaluation indexes, which is conducive to analysis from different angles, avoid over reliance on a single indicator (Zhang and Niu, 2013).

We ran Amos 22.0 software on the survey data and verified the research hypothesis with a structural equation model. Table 3 lists the specific path relationship of the research hypothesis model, which shows that the test results support the original hypothesis. Among them, the standardized path coefficient of emotional intelligence to surface behavior is 0.612, *t* = 4.329, It shows that under the OTA service recovery scenario, the emotional intelligence of service recovery employees has a significant positive impact on their surface behavior, providing support for H1. In OTA service recovery practice, employees with high emotional intelligence can better control and manage their emotions and their surface behaviors, such as smiles, expressions, etc. The standardized path coefficient of emotional intelligence on deep behavior is 0.693, *t* = 6.508, which verifies that the emotional intelligence of OTA employees has a positive and significant impact on their deep behavior, providing support for H2. The results of this study show that employees with high emotional intelligence are better at changing their thoughts and emotions, so as to make their inner cognition consistent with the work emotion required by the organization. These results also show that under the OTA service recovery scenario, emotional intelligence has a more significant impact on employees’ deep behavior than on their surface behavior.

**TABLE 3:** Hypothesis and testing.

Hypothesis	Structural path	Standard coefficients	*t*-value	Results
H1	Emotional intelligence → Surface behavior	0.612**	4.329	Supported
H2	Emotional intelligence → Deep behavior	0.693***	6.508	Supported
H3	Emotional intelligence → Service recovery satisfaction	0.375*	1.989	Supported
H4	Surface behavior → Service recovery satisfaction	0.209	1.034	Not Supported
H5	Deep behavior → Service recovery satisfaction	0.783***	7.373	Supported
H6	Service recovery satisfaction → Customer loyalty	0.835***	9.163	Supported

*p < 0.05, **p < 0.01, ***p < 0.001.

Table 3 shows that the relationship between emotional intelligence and customer satisfaction with service recovery is positive and significant, and its standardized path coefficient is 0.375, *t* = 1.989,providing support for H3. While the emotional intelligence of employees has a significant positive impact on service recovery satisfaction under the OTA service recovery scenario, the standardized path coefficient is low, and the effect is limited. Table 3 also shows that the standardized path coefficient of surface behavior and service recovery satisfaction is 0.209, but *t* = 1.034, which is not statistically significant, providing support for H4. The standard path coefficient of deep behavior to service recovery satisfaction is 0.783, *t* = 7.373, providing support for H5. The research results of H4 and H5 show that under the OTA service recovery scenario, employees’ surface behavior will not reduce service recovery satisfaction, but the positive impact on service recovery satisfaction is not significant, while deep behavior can increase service recovery satisfaction. Therefore, in service recovery, we should pay more attention to the deep behavior of service recovery employees. Table 3 also shows that the standardized path coefficient of service recovery satisfaction to customer loyalty is 0.835, *t* = 9.163, providing support for H6. It shows that under the OTA service recovery scenario, service recovery satisfaction is a direct influencing variable of customer loyalty, and service recovery satisfaction is a necessary preparation for customer loyalty.

### Mediating Effect Test

This study uses Bootstrap method to test the mediating effect. This is because in the simple mediation test, the bootstrap method has obvious advantages and will be more scientific and accurate than the causal stepwise regression method (Preacher et al., 2007). At the same time, the sampling in this study does not conform to the normal distribution, and the Bootstrap method does not need to assume the normal distribution of the sampling. It estimates the indirect effect and sampling distribution through repeated sampling, and estimates the confidence interval of the indirect effect according to the distribution characteristics (Preacher et al., 2007; Wu, 2010).

In order to further confirm the existence of a mediation effect, we constructed a competition model and a mediation model. Table 4 shows the goodness-of-fit indicators of the competition model and the mediation model. According to Wu (2010), the criteria for a good model are that χ^2^/DF is less than 5, CFI and TLI are greater than 0.90, SRMR is less than 0.05, and RMSEA is less than 0.1. The χ^2^/DF of the competition model was 5.791, and other indicators did not meet the requirements, indicating that the model was not well fitted. However, χ^2^/DF, CFI, TLI, SRMR CFI, TLI, SRMR, and RMSEA of the chain intermediary model all met the requirements and were better than those of the competition model. Therefore, a mediation effect does exist.

**TABLE 4:** Test of the competition model for the mediating effect.

Model	χ^2^	DF	χ^2^/DF	CFI	TLI	SRMR	RMSEA
Chain mediating effect	47.546	18	2.641	0.932	0.923	0.040	0.062
Parallel mediating effect (competition model)	139.963	24	5.791	0.798	0.843	0.065	0.109

The mediation effect results are shown in Table 5. The indirect effect value of the intermediary path “Emotional intelligence → Service recovery satisfaction → Customer loyalty” was 0.244, accounting for 40.87% of the total effect. It is confirmed that service recovery satisfaction plays a mediating effect between emotional intelligence and customer loyalty, providing support for H7. It shows that in the OTA service recovery scenario, employees’ emotional intelligence will indirectly affect customer loyalty, but it needs the mediating effect of service recovery satisfaction. Therefore, improving employees’ emotional intelligence level is an important way to improve customer loyalty. The indirect effect value of the mediating path “Emotional intelligence → Surface behavior → Service recovery satisfaction → Customer loyalty” was 0.101, accounting for 16.92% of the total effect, indicating that surface behavior and service recovery satisfaction play mediating roles in the relationship between emotional intelligence and customer loyalty, providing support for H8. Meanwhile, the indirect effect value of the mediating path “Emotional intelligence → Deep behavior → Service recovery satisfaction → Customer loyalty” was 0.126, accounting for 21.10% of the total effect, indicating that deep behavior and service recovery satisfaction play mediating roles in the relationship between emotional intelligence and customer loyalty, providing support for H9. The mediating effect test results of H8 and H9 show that in the OTA service recovery scenario, in the relationship between emotional intelligence and customer loyalty, surface behavior, deep behavior and service recovery satisfaction play a mediating effect. Therefore, in addition to improving employees’ emotional intelligence level, we should also improve employees’ emotional labor (surface behavior and deep behavior) to improve service recovery satisfaction and customer loyalty. As shown in Table 5, the confidence intervals of the total mediating effect and the three mediating effects did not contain 0, which meant they were statistically significant. The specific test results are shown in Table 5.

**TABLE 5:** Test results for mediating effects.

Mediating effect path	Indirect effect value	Standard error boot SE	Upper limit boot CI	Lower limit boot CI	Effect proportion
1. Emotional intelligence→Service recovery satisfaction→Customer loyalty	0.244	0.021	0.216	0.443	40.87%
2. Emotional intelligence→Surface behavior→Service recovery satisfaction →Customer loyalty	0.101	0.036	0.054	0.109	16.92%
3. Emotional intelligence→Deep behavior→Service recovery satisfaction→Customer loyalty	0.126	0.009	0.071	0.146	21.10%
4. Total mediating effect	0.471	0.021	0.359	0.601	78.89%
5. Total effect	0.597	0.013	0.420	0.793	100%

## Conclusion and Discussion

### Conclusion and Theoretical Contributions

First, under the OTA service recovery scenario, the emotional intelligence of employees has a significant positive impact on their surface behavior and deep behavior, and the influence on the latter is stronger and more significant. This shows that OTA employees with higher emotional intelligence are better at emotional labor (surface behavior and deep behavior), and emotional intelligence has a greater impact on deep behavior than on surface behavior. This finding is consistent with that of Totterdell and Holman (2003); furthermore, Chen et al. (2010) found that emotional intelligence has a positive impact on emotional labor (surface behavior and deep behavior) and further verified the direct impact of employees’ emotional intelligence on emotional labor (surface behavior and deep behavior) under the OTA service recovery scenario, thus deepening and expanding the connotation and applicability of the influence relationship between emotional intelligence and emotional labor.

Second, under the OTA service recovery scenario, the emotional intelligence and deep behavior of employees have a positive impact on service recovery satisfaction, while surface behavior has a non-significant positive effect on service recovery satisfaction. This finding does not support H4. The results indicate that employees with high emotional intelligence and good deep behavior will elicit higher satisfaction with service recovery. Previous studies found that the emotional intelligence of salesmen and cashiers has a positive impact on sales performance and customer satisfaction (Brown and Sulzer-Azaroff, 1994; Rozell et al., 2006); this study confirmed that the emotional intelligence of employees in an OTA service recovery scenario has a positive impact on service recovery satisfaction, which enriches the research on emotional intelligence and provides an empirical reference for further analysis of the influencing factors of service recovery satisfaction. At the same time, our findings are not entirely consistent with Zhang’s (2011) conclusion that surface behavior causes customer aversion and a sincere display can significantly improve customer satisfaction, indicating that surface behavior does not necessarily cause customer aversion, thus deepening the understanding of the relationship between emotional labor and service satisfaction and expanding its application scenarios.

Third, under the OTA service recovery scenario, service recovery satisfaction has a positive impact on customer loyalty. Surface behavior, deep behavior, and service recovery satisfaction play mediating roles in the relationship between emotional quality and customer loyalty. The finding that service recovery satisfaction has a positive impact on customer loyalty further confirms the research results of Lu (2007), Yu et al. (2008) and extends the research to the OTA service recovery scenario, expanding the applicable scenarios and research on service recovery satisfaction and customer loyalty.

## Practical Applications

Firstly, the research results have important guiding significance for employee behavior in service environment. According to the results of this study, OTA employees’ emotional intelligence has a significant positive impact on emotional labor (surface behavior and deep behavior) and service recovery satisfaction. Therefore, employees in the service environment should strive to improve their emotional intelligence and customer satisfaction. In service work, especially in service recovery work, employees should pay attention to controlling and managing their emotions and reducing customers’ negative emotions. For example, in the case of service failure, when encountering angry customers, employees should try their best to empathize, think more about customers, control their emotions, do not speak words to stimulate customers, and do not complain about customers. Otherwise, the event of service failure will be enlarged or even uncontrollable. In terms of language communication, employees should sincerely apologize to customers, communicate with them in a language that respects customers, and patiently listen to their service recovery demands. In this process, employees also need to ease the negative emotions of customers and impress customers with sincerity.

Second, OTA should pay more attention to the role of employees’ deep behavior, although the surface behavior cannot be ignored. Our findings show that employees’ deep behavior has a positive impact on service recovery satisfaction, while surface behavior has no significant negative impact on service recovery satisfaction. Deep behavior is an honest display of emotions, which can convey honest and cordial information to customers and help the employees respond positively to customers. Under the OTA service recovery situation, customers can feel that they have lost “face,” especially in Chinese culture. Therefore, employees’ surface behavior cannot be ignored. Employees should display appropriate surface behavior to maintain customers’ “face.” Although customers can detect the false emotional display of employees’ surface behavior, due to the awareness of “face,” customers receiving service recovery do not hate such emotional expression. Enterprises should require tourism service staff to provide “service with a smile” in their work, try to please customers, and be good at showing positive emotions.

Third, employees’ emotional intelligence and emotional labor (surface behavior, deep behavior) cannot directly promote the formation of customer loyalty, but it can indirectly affect customer loyalty through service recovery satisfaction. Therefore, OTA must ensure the satisfaction of service recovery so that customers continue to patronize and recommend them to their relatives and friends after service recovery. Emotional intelligence and emotional labor are only important incentives to enhance customer loyalty, not direct factors. Therefore, in OTA service recovery practice, we should pay attention to the creation of service recovery satisfaction. OTA and hotels should optimize the recovery process and service recovery strategy, adopt scientific remedial methods to calm customer dissatisfaction, be good at approaching customers, and solve problems and difficulties for customers. At the same time, we should publicize the convenience and preferential measures of OTA, expand the reputation of OTA, and improve the satisfaction of service recovery.

## Research Limitations and Prospects

First, situational experiment has some limitations. The purpose of this study is to use the situational experiment method for empirical analysis. The experiment of this study simulated three scenarios of OTA service recovery, and all subjects were randomly divided into three groups, each set in one scenario. In each group, 50% of the tested objects act as OTA service recovery employees and 50% of the tested objects act as OTA customers. Due to the limitation of time and space, it is difficult for tourism researchers to investigate and study on service failure and service recovery. Therefore, the situational experiment method is very suitable for collecting customer data, and its internal validity is high. However, from the perspective of the situational experiment design of this study, the data of the situational experiment come from the subjective evaluation of customers, and the subjective evaluation is often biased due to the influence of mood and experience. In addition, in the scenario experiment, this study allows 50% of the subjects to play employees and 50% of the subjects to play customers. Whether they can completely fill in the questionnaire from the standpoint of employees or customers is also a factor to be considered. Therefore, in the future research, we will collect the objective data of customers at the same time. Objective data mainly refers to the data generated by customers using OTA platform, such as customer login time and times on OTA platform, amount consumed on OTA platform, customer service failure, etc. In future research, we will combine objective data and subjective data to improve the effectiveness and scientificity of the research.

Second, we need to extend the research model. This study explored the relationship between emotional intelligence, emotional labor and customer loyalty after service recovery under OTA service recovery scenario, but did not explore the relationship between customer forgiveness and customer emotion on related variables. Previous studies lacked attention, so there was a “theoretical black hole.” For example, in OTA service recovery practice, customer forgiveness is an important factor in the formation of service recovery satisfaction and loyalty. In the future research, we will incorporate customer forgiveness, customer emotion and other variables into the research model to better explore the influencing factors and formation process of OTA service recovery effect.

Third, we need to further study the influence of each dimension of emotional intelligence. This study took emotional intelligence as a variable and did not divide it into dimensions. Some scholars believe that emotional intelligence can be divided into different dimensions, such as emotional perception and emotional management (Salovey and Mayer, 1990; Mayer and Salovey, 2004). Therefore, in the future research of OTA service recovery, we should conduct in-depth research on the relationship between each dimension of emotional intelligence and related variables, enrich the theoretical accumulation of emotional intelligence and service recovery research, which will have greater guiding value for OTA business practice and promote the healthy development of OTA business model.

## Data Availability Statement

The original contributions presented in the study are included in the article/supplementary material, further inquiries can be directed to the corresponding author/s.

## Ethics Statement

Ethical review and approval was not required for the study on human participants in accordance with the local legislation and institutional requirements. Written informed consent from the participants was not required to participate in this study in accordance with the national legislation and the institutional requirements.

## Author Contributions

JW was responsible for proposing research concepts, research hypotheses, scenario experiments and data analysis, and wrote and revised the manuscript. ZH participated in the scene experiment and manuscript revision. XZ participated in data analysis and manuscript revision. All authors contributed to the article and approved the submitted version.

## Conflict of Interest

The authors declare that the research was conducted in the absence of any commercial or financial relationships that could be construed as a potential conflict of interest.

## Publisher’s Note

All claims expressed in this article are solely those of the authors and do not necessarily represent those of their affiliated organizations, or those of the publisher, the editors and the reviewers. Any product that may be evaluated in this article, or claim that may be made by its manufacturer, is not guaranteed or endorsed by the publisher.
